# Supplementary material for: Patterns of Intron Gain and Loss in Fungi
Source: PLoS Biol. 2004 Nov 30;2(12):e422. doi: 10.1371/journal.pbio.0020422 (PMC532390; doi:10.1371/journal.pbio.0020422)
Supplement: Table S1 — Also available at http://genes.mit.edu/NielsenEtAl/. (4.3 MB ZIP). [file pbio.0020422.st001.zip › NielsenEtAl/html/663.html]

AN0819.1.NCU02599.1.MG01208.1.FG00898.1


```
 CLUSTAL W (1.82) Multiple Sequence Alignments - Introns Inserted


Sequence 1: NCU02599.1	212 aa
Sequence 2: FG00898.1	213 aa
Sequence 3: MG01208.1	212 aa
Sequence 4: AN0819.1	214 aa
Alignment Length: 217 aa
Number Identitical Residues: 108 aa
Alignment Score (without introns) 5225


MG01208.1 	MTLYYTL0VFVLLMAEMGLFMLLIVPLPFTIRRRMFT2FISENPLVAKVQYAMK~ITFIF
NCU02599.1	MTLYYSL0VFMLLVAEMSIFMLLIVPLPFTIRRRLFT~FISENPIIAKLQYGLK~ITFIF
FG00898.1 	MTLYYTL0VFMLLVFEMGMFVLLIFPMPFGVRRKIFT2FISENPVVAKIQYWMK~ITFIF
AN0819.1  	MTLYYSL0VFCLLVLEMGVFMGLIVPLPFTVKRKLFT~FISESPVIAKLQYGLR0ITFIF
          	*****:* ** **: **.:*: **.*:** ::*::** ****.*::**:** :: *****

MG01208.1 	ILILFIDSVNRVYRVQLELVAASENNK--NA2PTIMGHERLEVQARKFYSQRNMYLCGFT
NCU02599.1	ILILFIDSVNRVYRVQVELAAATDASK-GNA2AAIMGHERLEVQARKFYSQRNMYLCGFT
FG00898.1 	ILILFVDSVNRVYRVQLELAAASEQAKHGGG2AAVMGHERLEVQARKFYSQRNMYLCGFT
AN0819.1  	ILILFIDSVNRVYRVQLEVSAFSKEGG-NVG2GAALGTDRMEVQARKFYSQRNMYLCGFT
          	*****:**********:*: * :.    . .  : :* :*:*******************

MG01208.1 	LFLSLILNRTYTMILDVLRLEEKVKQYEGTDKSHSAGKQSAKLAEAGDAGEIARLKKELE
NCU02599.1	LFLSLILNRTYIMILEVLRLEEKLKKFEGTDKDT---KQSEKLAMAGDPGEISRLKREIQ
FG00898.1 	LFLSLILNRTYVMIIEVMRLEDKVRSYEGTKDNT---KEAEKLAAAGKPGELARLRKELE
AN0819.1  	LFLSLILNRTYTMILETLRLEDRVKLLEGNKQAG--GKDSARLAAAGDMGEIGRLKKELE
          	*********** **::.:***::::  **...    .*:: :** **. **:.**::*::

MG01208.1 	RKEKNIQALKTQSEGLHREYDSLGEKF--TVQDGSNKKER-
NCU02599.1	LRDQDIETLKKQSASLHREYDELAEKYGRTQQDDVPKKMK-
FG00898.1 	LKEQDLKTLKKQSEQLHKSYDELSDKYAATQQTGEGRKGI-
AN0819.1  	EKERDIETLKKQCEGLTREYHKLGDQVTGKKSDDSVEKKDL
          	 :::::::**.*.  * :.*..*.::   . . .  .*
```
